# Seed priming with selenium: Effects on germination, seedling growth, biochemical attributes, and grain yield in rice growing under flooding conditions

**DOI:** 10.1002/pld3.378

**Published:** 2022-01-20

**Authors:** Feng‐qin Hu, Shuo‐chen Jiang, Zhun Wang, Kang Hu, Yi‐mei Xie, Ling Zhou, Jian‐qiang Zhu, Dan‐ying Xing, Bin Du

**Affiliations:** ^1^ College of Agriculture Yangtze University Jingzhou China; ^2^ Shoufu Engineering Design Company Hubei Branch Wuhan China; ^3^ National Quality Supervision and Inspection Center of Selenium Rich Products Enshi China

**Keywords:** anaerobic sprouting, direct‐seeded rice, flooding stress, selenium seed priming, starch hydrolysis efficiency

## Abstract

Prevalent irregular rainfall, flooding for weed control, and unleveled fields in the middle and lower reaches of the Yangtze River all contribute to flooding stress on germination and growth of direct‐seeded rice ( *Oryza sativa* L.). Herein, some experiments were conducted so as to assess the effects of seed priming with selenium (Se) on the germination and growth of rice under hypoxia. The experiment was arranged in a completely randomized factorial design with two factors and five replicates. Factors included Se concentration (0, 30, and 60 μmol/L) and duration of flooding stress (0, 2, 4, and 8 days). The experimental results showed that Se accelerated seed germination and increased emergence index and final emergence percentage. Additionally, Se increased shoot and root lengths and dry weights, but high Se concentration (60 μmol/L) reduced 18‐day‐old seedling dry weight under long‐term flooding (8 days). Furthermore, Se reduced malondialdehyde content and increased starch hydrolysis efficiency in seeds, superoxide dismutase, peroxidase, catalase, and glutathione peroxidase activities and seedling soluble protein and total chlorophyll contents. Se improved seedling total Se and organic Se contents while increasing total dry weight and yield. Notably, the highest yield was obtained after a 4‐day flooding period. Although Se priming favored rice seedling emergence and growth under flooding conditions, Se concentrations equal or above 60 μmol/L increased the risk of seedling death during long‐term flooding (≥8 days).

## 1 INTRODUCTION

Rice (*Oryza sativa* L.) is one of the most important staple cereal crops in the world, and more than half of the population in China depends on rice for food (Kennedy, 2002). With the acceleration of industrialization and urbanization, the labor force engaged in agricultural production in China is rapidly shifting to secondary and tertiary industries, leading to a rapid rise in the costs of agricultural labor (Lu et al., 2019). The traditional system of rice production in China, which involves the transplanting of seedlings from a nursery into a paddy field, faces unprecedented challenges because the production pattern is labor, water, and energy intensive, making the overall process less profitable (Ge et al., 2018). As an alternative, and owing to its low‐cost and labor‐saving features, direct seeding is currently receiving much attention worldwide and is being widely promoted, especially in China. Direct seeding contributes less greenhouse gas emissions than rice transplanting, thereby contributing to environmental protection efforts and sustainable agricultural development (Tao et al., 2016).

However, when direct seeding is subjected to heavy precipitation or unleveled fields during seed germination and seedling growth, rice seeds become susceptible to flooding (Lal et al., 2018). Moreover, weeds are more competitive than rice seeds and may severely inhibit the growth of directly seeded rice seedlings (Chamara et al., 2018). Fortunately, soil flooding after direct seeding is an effective, environmentally friendly, and low‐cost weed control method (Chamara et al., 2018). Nevertheless, irregular rainstorms, unleveled fields, or flooding for weed prevention may cause hypoxia (low O_2_ availability) or anoxia (no O_2_) stress in rice seedlings. Although rice is the only cereal crop that can germinate and extend its coleoptile under oxygen‐limited conditions (Ismail et al., 2009), an insufficient oxygen supply will inhibit the aerobic respiration of rice, causing carbohydrates stored in the endosperm to provide only a small amount of energy through oxidation pathways to support the elongation of the coleoptile. Consequently, rice seedlings may fail to develop roots and leaves (Ismail et al., 2009). Limiting oxygen may induce restricted seedling growth or death, the formation of uneven and insufficient production groups, and, eventually, reduced grain yields (Lal et al., 2018). Rice seedlings resist flooding mainly through two mechanisms: (1) the low‐oxygen quiescence syndrome, whereby the rice shoot does not elongate upon submergence but regrows after de‐submergence, or (2) low‐oxygen escape syndrome, whereby the shoot extends rapidly under flood waters to reach the water surface (Ma et al., 2020).

Selenium (Se) is an essential trace element for maintaining the normal functioning of many physiological processes (Kieliszek & Błażejak, 2016; Pappas et al., 2019). All forms of life, from primitive cells to complex organisms, require certain amounts of Se to be incorporated to special enzymes and cellular components for their metabolic functions (Oraby et al., 2015; Rayman, 2012). Studies have shown that low concentration of Se is beneficial for plant growth and development (Feng et al., 2013; Kaur et al., 2014). A small amount of Se can not only improve the quality and yield of plants but also modulate multiple stress‐responsive genes (Gupta & Gupta, 2017; Moulick et al., 2016; Wang et al., 2017). Khaliq et al. (2015) found that when soaking rice seeds with pure Se content between 15 and 60  μmol/L, the germination potential and germination rate of seeds were improved, as well as the activities of various enzymes. In vivo, antioxidative effect is one of the most important physiological functions of Se (Misra et al., 2015). Se can effectively improve the activity of antioxidant enzymes, reduce oxidative damage, and promote plant growth (Filek et al., 2008; Pedrero et al., 2008). Further, Se favors rice seedling emergence and seedling quality (Lidon et al., 2018).

Although studies on the effects of flood conditions or selenium on rice performance have been widely reported, still rarely study has been accomplished about the effects of Se on seed germination and seedling growth under flooding conditions. Herein, we hypothesized that seed priming with Se ensures the uniformity of germination and enhances seedling growth under limited‐oxygen conditions. Therefore, our objective was to investigate the effectiveness of Se application as a seed germination initiator under flooding conditions.

## 2 MATERIALS AND METHODS

The experiment was laid out in a split‐plot design with flooding duration (FD) as the main plot and Se concentration as the subplot. FD at four levels and Se at three levels were tested with five replicates, and 60 subplots were established in pots (Experiment 1) and in the field (Experiment 2). The FD levels comprised flooding with 10  cm (Ella et al., 2011; Sarkar, 2012) of water for 0 (FD_0_), 2 (FD_2_), 4 (FD_4_), or 8 d (FD_8_) after rice sowing. Se levels comprised dry rice seeds primed by immersion in a sodium selenite (Na_2_O_3_Se) solution at Se 0 (Se_0_), 30 (Se_30_), or 60  μmol/L (Se_60_).

In each treatment, after the rice seeds were disinfected with 15% NaClO for 15 min and rinsed with distilled water for 20 min, 25 g of seeds was placed in a 200‐ml conical flask containing a 125 initiator solution. The flask was placed in an incubator in darkness, at 25 ± 1°C and 80% relative humidity. After 24 h, the seeds were filtered through gauze, placed in distilled water for 20 min, rinsed five times with ultrapure water, and set aside. Autoclavable glass Petri dishes lined with double layers of filter paper were placed on a laboratory bench and left to air‐dry for 24 h.

The soil used in Experiment 1 was a silty clay loam with 24% sand (2.00–.02 mm), 40% silt (.02–.002 mm), and 36% clay (<.002 mm), collected from the plow layer (0–20 cm) of an arable field in Yangtze University, Hubei Province, Southern China. Each kilogram of soil at pH 5.94 contained 34.52 g of organic matter, 224.19 mg of available N, 1.37 mg of available P, 127.24 mg of available K, and .29 mg of total Se. The soil was air‐dried, sieved to <5 mm, and homogeneous. Basal fertilizers (120 mg N/kg soil as NH_4_NO_3_, 30 mg P/kg soil, 75.5 mg K/kg soil as K_2_HPO_4_) were added to the soil and mixed thoroughly. The mixed soil (5 kg) was packed into plastic pot with a diameter of 25 cm in diameter and a height of 30 cm. One hundred seeds were evenly sprinkled on the soil surface in each treatment, then immediately irrigated water by 10 cm, and maintained at a constant water level during inundation. After flooding duration ended, all treatments maintained a 1 cm water level until harvest (18 days). The FD were randomly arranged on a frame inside a glasshouse at 28/25°C day/night and a 16 h/day photoperiod with natural sunlight supplemented with sodium vapor lamps to maintain a light intensity > 350 μmol m^−2^ s^−1^.

Experiment 2 was conducted in 2018–2019 at the Yangtze University farm in Jingzhou County, Hubei Province, China (112°04′–112°05′N, 30°32′–30°33′E). The soil was the same as that used in Experiment 1. After land preparation, seeds treated in the same manner as those in Experiment 1 were sown directly in the field at a rate of 60 kg ha^−1^, followed immediately by 10‐cm‐high flooding. After flooding duration ended, the guidelines for the local high‐yield field water management mode were utilized. Each plot was fertilized as follows: N 150 kg ha^−1^, P_2_O_5_ 59 kg ha^−1^, and K_2_O 120 kg ha^−1^ applied in the form of CO (NH_2_)_2_, (NH_4_)_2_HPO_4_, and KCl, respectively. Specifically, 60% of N was applied as a basal fertilizer and the remaining 40% as a tillering fertilizer, whereas 100% of P_2_O_5_ and K_2_O were applied as base fertilizers. Treatments were arranged in a randomized complete block design with five replications and a plot area of 24.0 m^2^ (6 × 2 m). Manual weeding was performed in the early tiller, late tiller, and heading stages. Pest and disease incidence were intensively controlled.

### 2.1 Rice emergence

In Experiment 1, seedling emergence was counted daily using the method prescribed by the Association of Official Seed Analysts until the 10th day. A seedling was scored as ‘emerged’ when its hypocotyl length was ≥2  mm. The time to start emergence (TSE) of seeds were recorded. The time taken to 50% emergence (E_50_), mean emergence time (MET), emergence index (EI), and final emergence percentage (FEP) of seeds were calculated as follows (Khaliq et al., 2015):

(1) $$E_{50}=\mathrm{ti}+\frac{\left(\frac{N_{10}}{2}-\mathrm{ni}\right)\left(\mathrm{tj}‐\mathrm{ti}\right)}{\mathrm{nj}‐\mathrm{ni}}$$

where *N* is the number of emerged seeds in 10  days and *ni* and *nj* are the cumulative numbers of emerged seeds by adjacent counts at time *ti* and *tj*, respectively (*ni*  <  *N*/2  <  *nj*).

(2) $$\mathrm{MET}=\frac{\sum Dn}{\sum n}$$

where *n* is the number of seeds emerged on day *D* and *D* is the number of days counted from the beginning of emergence, *D*  ≤  10.

(3) $$\mathrm{EI}=\sum \frac{\mathrm{Et}}{\mathrm{Dt}}$$

where *Et* is the number of seeds emerged on day *t* and *Dt* is time corresponding to *Et*, 1  ≤  *t*  ≤  10.

(4) $$\mathrm{FEP}=\frac{E18}{\text{Number of}\;\mathrm{all}\;\text{seeds}}\times 100$$

where E18 is the number of emerged seeds in the 18th day.

### 2.2 Seedling morphology

In Experiment 1, shoot and root lengths of five randomly selected seedlings were measured both 10 and 18 days after sowing from each experimental unit in normally emerging seedlings. Five measured seedlings were oven‐dried at 70°C for 72 h to get the dry biomass. Then, the dried samples were ground into powder to pass through a .15‐mm sieve and sealed in ziplock bags before Se analysis.

### 2.3 Biochemical analyses

In Experiment 1, lipid peroxidation in the rice seeds on the second day was determined from the malondialdehyde (MDA) content using the thiobarbituric acid method (Yang et al., 2014). The α‐amylase activity in ground rice seeds on the second day was measured according to a reported technique (Mahakham et al., 2017). Total soluble sugar and starch contents in the rice seeds on the second day were quantified according to Khaliq et al. (2015). The activities of superoxide dismutase (SOD) at 560  nm, catalase (CAT) at 240  nm, peroxidase (POD) at 470  nm, and glutathione peroxidase (GPx) at 340  nm in rice seedlings in the 18th day were determined according to the methods in Du et al. (2019). Soluble protein content in rice seedlings in the 18th day was quantified according to the method of Bradford (1976).

### 2.4 Yield and total dry weight

In Experiment 2, grain yields and total dry weight were measured at maturity by taking 5‐m^2^ plant samples at the center of each plot. Plant samples were separated from the filled grains and straw. Filled grains and straw were dried in an oven at 70°C to a stable weight and weighted, and grain yield was calculated at 14% moisture content. The grain was further processed into polished rice, and polished rice samples were ground into powder to pass through a .15‐mm sieve and sealed in ziplock bags before Se analysis.

### 2.5 Determination of Se concentrations

Contents of total Se and organic Se in the seedlings (Experiment 1) and polished rice (Experiment 2) were determined according to the method of Deng et al. (2017).

### 2.6 Statistical analyses

All experimental data are expressed as means ± standard errors (SE) of five replicates. The dates were subjected to the two‐way analysis (ANOVA) to determine the effects of flooding duration, Se treatments, and interaction between them, respectively. Significant differences between flooding duration and Se treatments among the same year were tested by Duncan's multiple range tests. The significance level was *p* < .05.

## 3 RESULTS

### 3.1 Rice emergence

Flooding duration, Se treatment, and their interaction significantly affected rice emergence, although the interaction had no significant effect on TSE (Table 1). Generally, as flooding duration increased, TSE and MET increased, whereas EI decreased. Further, E_50_ was highest at FD_4_ and FEP was highest at FD_2_. Additionally, as Se concentration increased, TSE, E_50_, and MET decreased, whereas EI and FEP increased. It is worth noting that when flooding duration was less than 8  days, EI and FEP increased with the rising Se concentration, but when it extended for 8  days, EI and FEP in Se_60_ were lower than those in Se_30_. The results above indicate that long‐term flooding limits seed germination and Se priming promoted seed germination; however, when flooding duration was 8  days, Se_60_ had a negative effect on seed germination.

**TABLE 1:** Effects of selenium (Se) concentration and flooding duration on rice emergence

FD	Se	TSE	E50	MET	EI	FEP
0	0	2.40 ± .55ab	3.62 ± 0.10f	4.40 ± .05e	18.71 ± .44ef	78.40 ± 1.82cd
0	30	1.60 ± .55cd	3.53 ± .05gh	4.07 ± .09g	21.80 ± 1.33bc	82.20 ± 3.83bc
0	60	1.40 ± .55d	3.46 ± .04h	3.96 ± .06h	24.55 ± 1.12a	88.20 ± 2.05a
2	0	2.40 ± .55ab	4.38 ± .05c	4.88 ± .06c	18.41 ± 1.35fg	84.60 ± 5.13ab
2	30	2.00 ± 0.00bc	3.71 ± .04e	4.33 ± .05e	2.66 ± 1.02cd	84.00 ± 5.20ab
2	60	2.00 ± 0.00bc	3.61 ± .05fg	4.19 ± .07f	22.61 ± 1.10b	87.60 ± 4.83ab
4	0	2.40 ± .55ab	4.54 ± .05b	5.03 ± .05b	17.35 ± .50g	83.00 ± 1.22abc
4	30	2.00 ± 0.00bc	4.71 ± .04a	5.23 ± .03a	17.19 ± 1.06g	83.60 ± 5.41abc
4	60	2.00 ± 0.00bc	4.41 ± .08c	4.77 ± .07d	19.87 ± .95de	88.20 ± 2.95a
8	0	2.60 ± .55a	4.59 ± .07b	5.15 ± .07a	14.60 ± .69h	72.00 ± 3.39e
8	30	2.00 ± 0.00bc	4.57 ± .07b	5.15 ± .14a	18.15 ± .52fg	84.00 ± 4.00ab
8	60	2.00 ± 0.00bc	4.10 ± 0.11d	4.68 ± .09d	17.32 ± .97g	75.20 ± 3.70de
F_value_	FD	3.259*	751.798**	499.034**	82.248**	14.712**
	Se	14.778**	173.038**	201.975**	78.226**	1.143**
	FD × Se	.704ns	47.411**	201.975**	7.033**	4.958**

*Note*: Means (*n* = 5) with different letters differ significantly at the 5% probability level based on Tukey's test.

Abbreviations: E_50_, time taken to 50% emergence; EI, emergence index; FD, flooding duration; FEP, final emergence percentage; MET, mean emergence time; ns, nonsignificance at *p* > .05; TSE, time to start emergence.

* Significant at *p* < .05.

** Significant at *p* < .001.

### 3.2 Seedling morphology

Flooding duration, Se treatment, and their interaction significantly affected seedling morphology, although the interaction had no significant effect on shoot length on the 18th day (Table 2). As flooding duration increased, its negative effects on the growth of shoots and roots gradually increased. Compared with FD_0_, shoot length, root length, and dry biomass in FD_8_ declined by 41.74%, 81.70%, and 79.21%, respectively, on the 10th day and by 35.90%, 48.06%, and 59.89%, respectively, on the 18th day. Shoot and root lengths and dry biomass increased with increasing Se concentration. Compared with Se_0_, shoot and root lengths and dry biomass of Se_60_ decreased by 37.36%, 18.18%, and 27.40%, respectively, on the 10th day and by 24.39%, 15.58%, and 27.41%, respectively, on the 18th day. Notably, Se_60_ significantly reduced the dry biomass of 18‐day‐old seedlings in FD_8_. These results indicate that flooding restricted seedling growth, whereas Se priming promoted seedling growth; however, Se_60_ reduced the dry biomass of 18‐day‐old seedlings in FD_8_.

**TABLE 2:** Effects of selenium (Se) concentration and flooding duration on rice morphology

FD	Se	Seedling shoot length (cm)		Seedling root length (cm)		Seedling dry biomass (g)	
		10th day	18th day	10th day	18th day	10th day	18th day
0	0	5.13 ± 0.2de	11.28 ± .31bc	5.52 ± .09c	1.37 ± 0.33b	.59 ± .01c	1.05 ± .03d
0	30	5.83 ± .26c	11.92 ± .64b	6.48 ± .07b	11.57 ± .50a	.66 ± .03b	1.21 ± .02b
0	60	6.48 ± 0.20a	13.54 ± .38a	6.80 ± .23a	12.08 ± .42a	.77 ± .02a	1.33 ± .03a
2	0	4.89 ± .29e	1.65 ± 1.10cd	4.23 ± .16e	9.42 ± .77c	.43 ± .02e	.91 ± .05e
2	30	5.71 ± .37c	11.42 ± .65bc	5.05 ± .35d	1.38 ± .50b	.52 ± .02d	1.05 ± .05d
2	60	6.13 ± .25b	13.30 ± 1.12a	5.06 ± .55d	9.96 ± .78bc	.58 ± .03c	1.16 ± .04c
4	0	3.85 ± .13g	9.23 ± .16e	3.11 ± .26f	7.79 ± .26d	.30 ± .02g	.73 ± .04f
4	30	4.08 ± .19g	1.30 ± .67d	3.28 ± .27f	8.38 ± .54d	.31 ± .01g	.72 ± .05f
4	60	5.36 ± .22d	11.33 ± .12bc	3.37 ± .19f	1.32 ± .52b	.38 ± .02f	1.19 ± .03bc
8	0	2.51 ± .22i	6.52 ± .52g	1.06 ± .05g	5.55 ± .27e	.14 ± .01h	.52 ± .03g
8	30	3.12 ± .19h	8.33 ± .50f	1.16 ± .07g	6.19 ± .19e	0.15 ± 0.00h	.51 ± .02g
8	60	4.53 ± .16f	8.70 ± .48ef	1.22 ± .04g	5.93 ± 0.20e	.13 ± .01h	.41 ± .03h
F_value_	FD	354.739*	147.663*	1235.500*	346.937*	2296.481*	1066.688*
	Se	224.398*	66.026*	38.621*	37.279*	2296.481*	191.334*
	FD × Se	6.577*	1.663ns	6.964*	8.220*	3.269*	74.146*

*Note*: Means (*n* = 5) with different letters differ significantly at the 5% probability level based on Tukey's test.

Abbreviations: FD, flooding duration; ns, nonsignificance at *p* > .05.

* Significant at *p* < .001.

### 3.3 Biochemical attributes of seeds

Se treatments significantly affected the biochemical attributes of rice seeds (Table 3). MDA and starch contents decreased, whereas soluble sugar content and α‐amylase activity increased with increasing Se concentration. MDA and starch contents in Se_60_ were 25.11% and 12.88% lower, respectively; however, α‐amylase activity and soluble sugar content in Se_60_ were 19.52% and 6.69% higher, respectively, compared with the Se_0_ treatment. These results indicate that Se priming reduced MDA content and accelerated starch hydrolysis.

**TABLE 3:** Effects of selenium (Se) concentration on biochemical attributes of rice seeds

Se	MDA	Starch	α‐Amylase	Sugar
	(nm/g seed)	(% DW)	(units^a^)	(% DW)
0	11.27 ± 1.44a	5.24 ± 3.50a	8.35 ± .95c	7.17 ± .36b
30	9.90 ± 1.87b	46.68 ± 4.41b	9.15 ± .96b	7.34 ± .53b
60	8.44 ± .57c	43.77 ± 2.51c	9.98 ± .32a	7.65 ± .49a
F_value_	2.442**	16.560**	2.635**	5.512*

*Notes*: One unit of the enzyme's activity is the amount of enzyme that released 1 μmol of maltose by 1‐ml original enzyme solution in 1 min. Means (*n* = 5) with different letters differ significantly at the 5% probability level based on Tukey's test.

Abbreviation: ns, nonsignificance at *p* > .05.

* Significant at *p* < .01.

** Significant at *p* < .001.

### 3.4 Biochemical attributes of seedlings

Flooding duration, Se concentration, and their interaction significantly affected seedling biochemical attributes, although the interaction had no significant effect on GPx activity (Table 4). Antioxidant enzyme activities first increased and then decreased with increasing flooding duration. SOD and POD activities were highest in FD_4_ and lowest in FD_0_. Furthermore, SOD and POD activities in FD_0_ were 14.63% and 26.15% lower, respectively, compared with FD_4_. In turn, CAT and GPx activities were highest in FD_2_ and lowest in FD_8_, whereas SOD and POD activities in FD_8_ were 25.74% and 19.20% lower, respectively, compared with FD_2_. Additionally, soluble protein and total chlorophyll contents decreased with increasing flooding duration. Thus, compared with FD_0_, soluble protein and total chlorophyll contents in FD_8_ increased by 35.51% and 44.72%, respectively. In contrast, SOD, POX, CAT, and GPx activities and soluble protein and total chlorophyll contents increased with increasing Se concentration. Thus, compared with Se_0_, these six physiological indicators increased by 55.53%, 129.00%, 225.68%, 9.72%, 75.86%, and 46.02%, respectively, in Se_60_. Se priming tended to increase antioxidant enzyme activities, as well as soluble protein and total chlorophyll contents, although the extent of such increase was different under different flooding duration treatments. In summary, short‐term flooding stimulated antioxidant enzyme activities. Conversely, long‐term flooding damaged the physiology of seedlings, but Se restored and in fact enhanced the physiology of seedlings under conditions of 0–8  days of flooding.

**TABLE 4:** Effects of selenium (Se) concentration and flooding duration on biochemical attributes of rice seedlings

FD	Se	SOD	POD	CAT	GPx	Protein	Total chlorophyll
		(units/g protein)	(μmol min^−1^ g^−1^ protein)	(μmol min^−1^ g^−1^ protein)	(μmol min^−1^ g^−1^ protein)	(mg/g FW)	(mg/g FW)
0	0	565.38 ± 19.80g	1.04 ± .03j	.66 ± .01i	152.18 ± 5.21fg	.27 ± .01f	3.32 ± .08f
0	30	663.74 ± 16.60cd	1.68 ± .05g	1.32 ± .03f	199.03 ± 15.05de	.36 ± .01c	4.32 ± .07b
0	60	673.99 ± 55.55cd	2.25 ± .09f	1.66 ± .04d	289.77 ± 13.82a	.44 ± .01a	5.06 ± 0.21a
2	0	612.50 ± 27.39ef	1.23 ± .05i	.73 ± .04i	176.08 ± 7.77ef	.24 ± .02g	3.23 ± .12f
2	30	682.47 ± 50.76cd	2.36 ± .08de	1.48 ± .07e	231.87 ± 10.83bc	.35 ± .01c	4.28 ± .22b
2	60	923.54 ± 49.13a	2.77 ± .05b	2.49 ± .07a	307.70 ± 15.62a	.41 ± .02b	5.01 ± .41a
4	0	587.32 ± 27.17fg	1.42 ± .05h	.68 ± .05i	122.54 ± 69.06g	0.20 ± .01h	2.79 ± .16g
4	30	708.49 ± 16.45c	2.41 ± .06d	1.21 ± .05g	211.88 ± 13.36cd	.30 ± .01e	3.73 ± .08d
4	60	933.46 ± 21.75a	2.90 ± .12a	2.24 ± 0.10b	280.34 ± 6.19a	.36 ± .01c	4.01 ± .25c
8	0	457.42 ± 6.40h	.93 ± .04k	.50 ± .03j	137.15 ± 5.96g	.16 ± .01i	2.09 ± 0.10h
8	30	645.79 ± 18.68de	2.30 ± .09ef	1.01 ± .02h	197.60 ± 11.11de	0.21 ± .01h	2.32 ± .12h
8	60	877.01 ± 44.14b	2.66 ± .07c	1.98 ± .13c	243.53 ± 16.61b	.32 ± .02d	2.61 ± .19g
F_value_	FD	41.400**	205.600**	123.638**	10.756**	264.445**	313.270**
	Se	400.710**	2458.852**	2686.850**	169.853**	712.536**	238.307**
	FD × Se	24.517**	26.346**	47.915**	1.805ns	4.282*	12.625**

*Note*: Means (*n* = 5) with different letters differ significantly at the 5% probability level based on Tukey's test.

Abbreviations: CAT, catalase; FD, flooding duration; GPx: glutathione peroxidase; nonsignificance at *p* > .05; POD, peroxidase; SOD, superoxide dismutase.

* Significant at *p* < .01.

** Significant at *p* < .001.

### 3.5 Effect of Se concentration in seed soaking solution on seedlings

Flooding duration and Se treatments considerably affected seedling total Se and organic Se contents, although the interaction between them significantly affected only organic Se content (Table 5). Total Se and organic Se contents decreased with increasing flooding time, being 5.53% and 25.23% lower in FD_8_, respectively, compared with FD_0_. Conversely, total Se and organic Se contents increased with increasing Se concentration; indeed, they increased by 2.407 and 1.454  mg  kg^−1^, respectively, in Se_60_, compared with Se_0_. Se content of polished rice ranged between .101 and .121  mg  kg^−1^ across treatments. These results indicate that flooding reduced the transport of Se from the seed to the growing seedling, whereas Se priming significantly increased Se content in seedlings.

**TABLE 5:** Effects of selenium (Se) concentration and flooding duration on rice seedlings

FD	Se	Total Se	Organic Se
		mg/kg	mg/kg
0	0	.165 ± .002d	.082 ± .002h
0	30	1.506 ± .068c	.828 ± .024e
0	60	2.653 ± .069a	1.746 ± .062a
2	0	.151 ± .003d	.071 ± .003h
2	30	1.499 ± .029c	.814 ± .061e
2	60	2.545 ± .084b	1.620 ± .044b
4	0	.130 ± .002d	.063 ± .004h
4	30	1.468 ± .048c	.725 ± .029f
4	60	2.481 ± .123b	1.446 ± .039c
8	0	0.101 ± .003d	.051 ± .003h
8	30	1.490 ± .059c	.664 ± .031g
8	60	2.494 ± 0.108b	1.271 ± .070d
F_value_	FD	5.326*	95.491**
	Se	6982.631**	95.491**
	FD × Se	1.622ns	32.201**

*Note*: Means (*n* = 5) with different letters differ significantly at the 5% probability level based on Tukey's test.

Abbreviations: FD, flooding duration; ns, nonsignificance at *p* > .05.

* Significant at *p* < .01.

** Significant at *p* < .001.

### 3.6 Total dry weight, yield, and Se content in polished rice

Flooding duration, Se concentration, and their interaction significantly affected total dry weight and yield in both years, but the interaction had no significant effect on Se content in polished rice in 2018 (Table 6). In 2018–2019, total dry weight and yield first increased and then decreased as flooding duration increased. Both total dry weight and yield were highest in FD_4_ and lowest in FD_0_. As a result, mean dry weight and yield in FD_0_ were 9.60% and 6.80% lower, respectively, compared with FD_4_. Both total dry weight and yield increased with increasing Se concentration. The corresponding mean values increased by 6.96% and 7.04%, respectively, in Se_60_, compared with Se_0_. However, the Se_60_ treatment group showed reduced total dry weight and yield in FD_8_ in both years. In summary, the highest yield was obtained under the 4‐day flooding treatment with Se priming.

**TABLE 6:** Effects of selenium (Se) concentration and flooding duration on total dry weight, yield, and Se content in Polish rice in 2018–2019

FD	Se	2018			2019		
		Total dry weight	Yield	Se content	Total dry weight	Yield	Se content
		t/ha	t/ha	mg/kg	t/ha	t/ha	mg/kg
0	0	1.30 ± .55bc	5.15 ± 0.21efg	.101 ± .003d	10.34 ± .66cde	4.93 ± 0.15f	0.108 ± .003g
0	30	9.85 ± .45cd	5.06 ± .12fgh	.106 ± .004c	10.44 ± 0.30cd	5.11 ± .07def	0.112 ± .003f
0	60	10.11 ± .40bc	5.30 ± .19cde	0.108 ± .003abc	10.35 ± .14cde	5.51 ± 0.21bc	.113 ± .002ef
2	0	10.28 ± .51bc	5.13 ± 0.11efg	0.109 ± .004abc	10.25 ± .62de	5.05 ± .14ef	.114 ± .003def
2	30	10.66 ± .26b	5.37 ± 0.15bcd	.110 ± .002abc	11.02 ± .48bc	5.61 ± .12ab	.123 ± .002a
2	60	12.01 ± .90a	5.50 ± .08bc	.111 ± .004ab	11.48 ± .88b	5.67 ± .16ab	0.118 ± .002bc
4	0	10.33 ± .68bc	5.16 ± .22efg	0.107 ± .002bc	10.81 ± .26bcd	5.22 ± .18de	.117 ± .002bcd
4	30	11.35 ± .42a	5.56 ± 0.15b	.106 ± .003c	11.40 ± .22b	5.80 ± .24a	.121 ± .004ab
4	60	11.67 ± .52a	5.77 ± 0.15a	0.107 ± .004abc	12.35 ± .55a	5.82 ± .14a	.116 ± .002cde
8	0	9.38 ± .17d	4.88 ± .16h	0.112 ± .002a	9.70 ± .57ef	5.13 ± .13def	.119 ± .002bc
8	30	9.92 ± .37bcd	5.25 ± .05def	0.108 ± .005abc	10.79 ± .63bcd	5.33 ± 0.21cd	.119 ± .003bc
8	60	9.59 ± .53cd	4.96 ± 0.15gh	.111 ± .003ab	9.49 ± 0.33f	4.98 ± .26ef	.116 ± .003cde
F_value_	FD	29.083^***^	25.523^***^	10.073^***^	24.981^***^	24.268^***^	23.480^***^
	Se	11.345^***^	21.269^***^	3.240^*^	10.276^***^	34.530^***^	11.232^***^
	FD × Se	5.355^***^	5.795^***^	1.876ns	5.905^***^	7.751^***^	3.488^**^

*Note*: Means (*n* = 5) with different letters differ significantly at the 5% probability level based on Tukey's test.

Abbreviations: FD, flooding duration; ns, nonsignificance at *p* > .05.

^*^ Significant at *p* < .05.

^**^ Significant at *p* < .01.

^***^ Significant at *p* < .001.

## 4 DISCUSSION

In Southern China, rice seedling quality is ensured by the wet direct‐seeding method, which involves soaking the seeds until the embryo breaks through the husk and leaks white spots (Liu et al., 2014). During the sowing period, in which the seeds are generally in the second phase of germination, anaerobic stress caused by rain, uneven fields, or flooding for weed control will cause anaerobic respiration to replace aerobic respiration, thereby reducing the efficiency of starch hydrolysis into soluble sugars (Ella et al., 2011). In the experiments reported herein, seed germination and seedling growth were restricted with increasing duration of flooding conditions (Tables 1 and 2), consistently with results of a previous study (Ella et al., 2011). Furthermore, except for Se_60_ in FD_8_, Se seed priming accelerated seed germination, increased EI and FEP, and promoted seedling growth (Tables 1 and 2). Reportedly, rice germination and growth are promoted by seed priming with low Se concentrations (Moulick et al., 2016) but are inhibited at high Se concentrations (Du et al., 2019); consistently, our data showed that Se_60_ reduced 18‐day‐old seedling dry weight in FD_8_ (Table 2). Although Se_60_ enhanced the anaerobic stress‐escape mechanism by accelerating seed germination and promoting shoot and root growth, shoot elongation reportedly consumes more endosperm nutrients and reduces seedling dry weight under prolonged flooding (Nishiuchi et al., 2012). These results indicate that Se seed priming at a high concentration entails certain risks in the case of prolonged flooding in the field.

The level of MDA, an important biochemical indicator of plant stress, increased significantly under conditions of extended flooding duration but decreased markedly with increasing Se concentration (Table 3). This indicates that seeds subjected to Se priming show higher antioxidant capacity and whole‐cell membranes to resist flooding stress damage. Flooding induces oxidative stress, which in turn induces an increase in the production of MDA (Gautam et al., 2014). Concomitantly, the ability of α‐amylase to hydrolyze starch into soluble sugars provides energy for coleoptile growth under limiting O_2_ conditions and is therefore directly related to the ability of rice to resist flooding stress (Vijayan et al., 2018). This study showed that Se application accelerated starch hydrolysis (Table 3) and, consistently, seed priming with Se reportedly increases the activity of α‐amylase (Khaliq et al., 2015). These findings verify the hypothesis that Se can strengthen the escape mechanism of seedlings from low oxygen‐stress damage by accelerating starch hydrolysis.

Antioxidant SOD, POX, CAT, and GPx activities play an important role in the active‐oxygen scavenging system of plants (Ella et al., 2011). In this study, SOD, POX, CAT, and GPx activities were lowest in the FD_8_ treatment. In contrast, SOD and POX activities were highest in FD_4_, and CAT and GPx activities were highest in FD_2_ (Table 4). In agreement with these findings, prolonged flooding duration can reportedly reduce antioxidant enzyme activity (Mondal et al., 2020). Additionally, slight waterlogging stress causes MDA accumulation in rice (Table 3), thereby stimulating the antioxidant enzyme defense system to produce more SOD and POX and maintain oxidative balance in rice cells (Candan & Tarhan, 2012), which likely explains why antioxidant activities were highest in the FD_4_ and FD_2_ treatment groups. Application of Se also increased the antioxidant enzyme activity level (Table 4). Soluble protein content, an important indicator of total plant metabolism, decreased with prolonged flooding duration but increased with increasing Se concentration (Table 4). These findings corroborate two previous reports (Du et al., 2019; Vijayan et al., 2018). As shown in Table 4, total chlorophyll content decreased with flooding duration but increased with increasing Se concentration. Flooding reportedly promotes the consumption of nonstructural carbohydrates, resulting in a decrease in total chlorophyll content (Gautam et al., 2014). Conversely, Se can improve total chlorophyll content and photosynthesis in rice (Zhang et al., 2014). Seed priming with low Se concentrations can also increase total chlorophyll content (Khaliq et al., 2015). These findings confirm our conclusion that seed priming with Se helps rice seedlings to restore growth under flooding stress.

During imbibition, rice seeds soaked in a Se solution absorb Se through the aleurone layer into the endosperm and embryo cells (Khaliq et al., 2015). Subsequently, inorganic Se in the cells is metabolically assimilated into organic Se by enzymes such as cysteine synthase (Liu et al., 2011). In our experiment, flooding reduced the conversion of selenite to organic Se (Table 5), likely because flooding limits the activity of some enzymes related to Se metabolism in rice cells. In contrast, Se application increased Se concentration in rice seedlings (Table 5), thus corroborating the results of two previous studies (Li et al., 2016; Wang et al., 2012). However, the Se content in polished rice after Se priming did not reach the standard for Se‐rich rice (Table 6). Therefore, the production of Se‐rich rice requires additional Se application.

According to the results of Experiment 1, it can be seen that the interaction between flooding duration and selenium treatments enhanced seed vigor and seed vigor was positively correlated with field performance in rice (Yamauchi & Winn, 1996). The conclusion was confirmed by the results of Experiment 2 (Table 6). Our research indicated that different effects of selenium concentration on total dry weight, yield, and Se content in polished rice (except 2018) at different flooding duration (Table 6). In general, with the extension of flooding duration, increasing Se level promoted and then inhibited rice yield. Among them, the highest seedling total dry weight and grain yield were recorded for the FD_4_ treatment group. This might be attributed that proper seed priming treatment (Farooq et al., 2006) and low level of Se (Khaliq et al., 2015) could improve seed germination percentage and germination index, thus promoting early germination and resulting in better seedling quality and higher paddy yield (Farooq et al., 2018). However, when flooding duration was extended to 8  days, rice seedling total dry weight and grain yield decreased, thus confirming that long‐term flooding caused damage to the rice plants. Furthermore, the prolonged flooding duration reduced antioxidant enzyme activity (Table 4), which might affect the antioxidant function of Se in seeds, thus affecting seedling growth and development. Fortunately, in addition to the lower grain yield in the Se_60_/FD_8_ combination treatment, overall, Se increased rice grain yield, which might be related to the improvement of seedling quality. According to Sadeghzadeh and Rengel's (2011) research on Zn seed priming, it can be inferred that improvement in grain yield with Se seed priming might also be attributed to the role of Se in activation of various enzymes. Se seed priming could provide a strong foundation for the plant, which lead to better plant growth and seed setting (Moulick et al., 2018). At present, it has been proved that soaking seeds with Zn could increase rice yield (Rehman et al., 2018; Zulfiqar et al., 2021), but there are still few studies on Se seed priming to improve rice yield. Hence, it can be hypothesized that improvement in grain yield is probably due to the interaction between flooding duration and selenium treatments, which could promote seed germination, improve emergence rate and better seedling stand production, and thus improved effective tillering and seed setting. In the future, it is necessary to further study the effects of the interaction between flooding duration and selenium on rice yield and grain selenium content in order to provide more guidance for Se‐rich rice planting in direct seeding fields.

## CONFLICT OF INTEREST

The authors declare that they have no known competing financial interests or personal relationships that might have influenced the work reported in this paper. The results/data/figures in this manuscript have not been published elsewhere, nor are they under consideration by any other publisher. The corresponding author has read *Food Policy*'s author responsibilities and submits this manuscript in accordance with these policies. All the material is owned by the authors, and/or no permissions were required. In addition, the manuscript has been revised by many of our colleagues, but if the editor believes that the manuscript still needs English editing services, we fully agree and will pay the relevant fees.

## AUTHOR CONTRIBUTIONS

F.Q.H. was the principal investigator who designed and implemented the research. S.C.J. wrote the English version of the manuscript. D.Y.X. provided guidance during the experimentation process. J.Q.Z. reviewed the final version of the manuscript prior to submission for peer review. B.D. oversaw the study and performed statistical analysis. W.Z. provided English guidance in the process of revising the manuscript. K.H. provided help in completing the experiment. Y.M.X. & L.Z. helped with selenium testing.

## Supporting information


**Table S1‐1.** Analysis of variance for effects of Selenium (Se) concentration and flooding duration on rice emergence. FD: flooding duration
**Table S1‐2.** Effects of Selenium (Se) concentration and flooding duration on rice emergence. FD: flooding duration. TSE: time to start emergence, E50: time taken to 50% emergence, MET: mean emergence time, EI: emergence index, FEP: final emergence percentage. Means (n = 5) with different letters differ significantly at the 5% probability level based on Tukey's test.
**Table S2‐1.** Analysis of variance for effects of Selenium (Se) concentration and flooding duration on rice morphology. FD: flooding duration
**Table S2‐2.** Effects of Selenium (Se) concentration and flooding duration on rice morphology. FD: flooding duration. Means (n = 5) with different letters differ significantly at the 5% probability level based on Tukey's test.
**Table S3‐1.** Analysis of variance for effects of Selenium (Se) concentration on biochemical attributes of rice seeds.
**Table S3‐2.** Effects of Selenium (Se) concentration on biochemical attributes of rice seeds. One unit of the enzyme's activity is the amount of enzyme which released 1 μmol of maltose by 1 mL original enzyme solution in 1 min. Means (n = 5) with different letters differ significantly at the 5% probability level based on Tukey's test.
**Table S4‐1.** Analysis of variance for effects of Selenium (Se) concentration and flooding duration on biochemical attributes of rice seedlings. FD: flooding duration
**Table S4‐2.** Effects of Selenium (Se) concentration and flooding duration on biochemical attributes of rice seedlings. FD: flooding duration. SOD: superoxide dismutase, POD: peroxidase, CAT: catalase, GPx: glutathione peroxidase. Means (n = 5) with different letters differ significantly at the 5% probability level based on Tukey's test.
**Table S5‐1.** Analysis of variance for effects of Selenium (Se) concentration and flooding duration on rice seedlings Se concentration. FD: flooding duration
**Table S5‐2.** Effects of Selenium (Se) concentration and flooding duration on rice seedlings Se concentration. FD: flooding duration. Means (n = 5) with different letters differ significantly at the 5% probability level based on Tukey's test.
**Table S6‐1.** Analysis of variance for effects of Selenium (Se) concentration and flooding duration on total dry weight, yield and Se content in polish rice in 2018–2019. FD: flooding duration
**Table S6‐2.** Effects of Selenium (Se) concentration and flooding duration on total dry weight, yield and Se content in polish rice in 2018–2019. FD: flooding duration. Means (n = 5) with different letters differ significantly at the 5% probability level based on Tukey's test.

## Data Availability

The datasets analyzed in this study are available from the corresponding author upon reasonable request.
