# Supplementary material for: Seed priming with selenium: Effects on germination, seedling growth, biochemical attributes, and grain yield in rice growing under flooding conditions
Source: Plant Direct. 2022 Jan 20;6(1):e378. doi: 10.1002/pld3.378 (PMC8776355; doi:10.1002/pld3.378)
Supplement: Supplementary file 1 — Table S1‐1. Analysis of variance for effects of Selenium (Se) concentration and flooding duration on rice emergence. FD: flooding duration Table S1‐2. Effects of Selenium (Se) concentration and flooding duration on rice emergence. FD: flooding duration. TSE: time to start emergence, E50: time taken to 50% emergence, MET: mean emergence time, EI: emergence index, FEP: final emergence percentage. Means (n = 5) with different letters differ significantly at the 5% probability level based on Tukey's test. Table S2‐1. Analysis of variance for effects of Selenium (Se) concentration and flooding duration on rice morphology. FD: flooding duration Table S2‐2. Effects of Selenium (Se) concentration and flooding duration on rice morphology. FD: flooding duration. Means (n = 5) with different letters differ significantly at the 5% probability level based on Tukey's test. Table S3‐1. Analysis of variance for effects of Selenium (Se) concentration on biochemical attributes of rice seeds. Table S3‐2. Effects of Selenium (Se) concentration on biochemical attributes of rice seeds. One unit of the enzyme's activity is the amount of enzyme which released 1 μmol of maltose by 1 mL original enzyme solution in 1 min. Means (n = 5) with different letters differ significantly at the 5% probability level based on Tukey's test. Table S4‐1. Analysis of variance for effects of Selenium (Se) concentration and flooding duration on biochemical attributes of rice seedlings. FD: flooding duration Table S4‐2. Effects of Selenium (Se) concentration and flooding duration on biochemical attributes of rice seedlings. FD: flooding duration. SOD: superoxide dismutase, POD: peroxidase, CAT: catalase, GPx: glutathione peroxidase. Means (n = 5) with different letters differ significantly at the 5% probability level based on Tukey's test. Table S5‐1. Analysis of variance for effects of Selenium (Se) concentration and flooding duration on rice seedlings Se concentration. FD: flooding duration Table S5‐2. Effec [file PLD3-6-e378-s001.docx]

**Table 1-1.** Analysis of variance for effects of Selenium (Se) concentration and flooding duration on rice emergence. FD: flooding duration

|  | TSE | | | E_50_ | | | MET | | | EI | | | FEP | | |
| --- | --- | --- | --- | --- | --- | --- | --- | --- | --- | --- | --- | --- | --- | --- | --- |
|  | df | F value | P value | df | F value | P value | df | F value | P value | df | F value | P value | df | F value | P value |
| FD | 3 | 3.259 | 0.0294 | 3 | 751.798 | 0.0000 | 3 | 499.034 | 0.0000 | 3 | 82.248 | 0.0000 | 3 | 14.712 | 0.0000 |
| Se | 2 | 14.778 | 0.0000 | 2 | 173.038 | 0.0000 | 2 | 201.975 | 0.0000 | 2 | 78.226 | 0.0000 | 2 | 10.143 | 0.0002 |
| FD × Se | 6 | 0.704 | 0.6480 | 6 | 47.411 | 0.0000 | 6 | 27.692 | 0.0000 | 6 | 7.033 | 0.0000 | 6 | 4.958 | 0.0005 |

**Table 1-2.** Effects of Selenium (Se) concentration and flooding duration on rice emergence. FD: flooding duration. TSE: time to start emergence, E50: time taken to 50% emergence, MET: mean emergence time, EI: emergence index, FEP: final emergence percentage. Means (n = 5) with different letters differ significantly at the 5% probability level based on Tukey's test.

| FD | Se | TSE | E_50_ | MET | EI | FEP |
| --- | --- | --- | --- | --- | --- | --- |
| 0 | 0 | 2.40±0.55ab | 3.62±0.10f | 4.40±0.05e | 18.71±0.44ef | 78.40±1.82cd |
| 0 | 30 | 1.60±0.55cd | 3.53±0.05gh | 4.07±0.09g | 21.80±1.33bc | 82.20±3.83bc |
| 0 | 60 | 1.40±0.55d | 3.46±0.04h | 3.96±0.06h | 24.55±1.12a | 88.20±2.05a |
| 2 | 0 | 2.40±0.55ab | 4.38±0.05c | 4.88±0.06c | 18.41±1.35fg | 84.60±5.13ab |
| 2 | 30 | 2.00±0.00bc | 3.71±0.04e | 4.33±0.05e | 20.66±1.02cd | 84.00±5.20ab |
| 2 | 60 | 2.00±0.00bc | 3.61±0.05fg | 4.19±0.07f | 22.61±1.10b | 87.60±4.83ab |
| 4 | 0 | 2.40±0.55ab | 4.54±0.05b | 5.03±0.05b | 17.35±0.50g | 83.00±1.22abc |
| 4 | 30 | 2.00±0.00bc | 4.71±0.04a | 5.23±0.03a | 17.19±1.06g | 83.60±5.41abc |
| 4 | 60 | 2.00±0.00bc | 4.41±0.08c | 4.77±0.07d | 19.87±0.95de | 88.20±2.95a |
| 8 | 0 | 2.60±0.55a | 4.59±0.07b | 5.15±0.07a | 14.60±0.69h | 72.00±3.39e |
| 8 | 30 | 2.00±0.00bc | 4.57±0.07b | 5.15±0.14a | 18.15±0.52fg | 84.00±4.00ab |
| 8 | 60 | 2.00±0.00bc | 4.10±0.11d | 4.68±0.09d | 17.32±0.97g | 75.20±3.70de |

**Table 2-1.** Analysis of variance for effects of Selenium (Se) concentration and flooding duration on rice morphology. FD: flooding duration

|  | Seedling shoot length (cm) | | | | | | Seedling root length (cm) | | | | | |
| --- | --- | --- | --- | --- | --- | --- | --- | --- | --- | --- | --- | --- |
|  | 10^th^ day | | | 18^th^ day | | | 10^th^ day | | | 18^th^ day | | |
|  | df | F value | P value | df | F value | P value | df | F value | P value | df | F value | P value |
| FD | 3 | 354.739 | 0.0000 | 3 | 147.663 | 0.0000 | 3 | 1235.500 | 0.0000 | 3 | 346.937 | 0.0000 |
| Se | 2 | 224.398 | 0.0000 | 2 | 66.026 | 0.0000 | 2 | 38.621 | 0.0000 | 2 | 37.279 | 0.0000 |
| FD × Se | 6 | 6.577 | 0.0000 | 6 | 1.663 | 0.1507 | 6 | 6.964 | 0.0000 | 6 | 8.220 | 0.0000 |

**Table 2-2.** Effects of Selenium (Se) concentration and flooding duration on rice morphology. FD: flooding duration. Means (n = 5) with different letters differ significantly at the 5% probability level based on Tukey's test.

| FD | Se | Seedling shoot length (cm) | | Seedling root length (cm) | | Seedling dry biomass (g) | |
| --- | --- | --- | --- | --- | --- | --- | --- |
|  |  | 10^th^ day | 18^th^ day | 10^th^ day | 18^th^ day | 10^th^ day | 18^th^ day |
| 0 | 0 | 5.13±0.2de | 11.28±0.31bc | 5.52±0.09c | 10.37±0.33b | 0.59±0.01c | 1.05±0.03d |
| 0 | 30 | 5.83±0.26c | 11.92±0.64b | 6.48±0.07b | 11.57±0.50a | 0.66±0.03b | 1.21±0.02b |
| 0 | 60 | 6.48±0.20a | 13.54±0.38a | 6.80±0.23a | 12.08±0.42a | 0.77±0.02a | 1.33±0.03a |
| 2 | 0 | 4.89±0.29e | 10.65±1.10cd | 4.23±0.16e | 9.42±0.77c | 0.43±0.02e | 0.91±0.05e |
| 2 | 30 | 5.71±0.37c | 11.42±0.65bc | 5.05±0.35d | 10.38±0.50b | 0.52±0.02d | 1.05±0.05d |
| 2 | 60 | 6.13±0.25b | 13.30±1.12a | 5.06±0.55d | 9.96±0.78bc | 0.58±0.03c | 1.16±0.04c |
| 4 | 0 | 3.85±0.13g | 9.23±0.16e | 3.11±0.26f | 7.79±0.26d | 0.30±0.02g | 0.73±0.04f |
| 4 | 30 | 4.08±0.19g | 10.30±0.67d | 3.28±0.27f | 8.38±0.54d | 0.31±0.01g | 0.72±0.05f |
| 4 | 60 | 5.36±0.22d | 11.33±0.12bc | 3.37±0.19f | 10.32±0.52b | 0.38±0.02f | 1.19±0.03bc |
| 8 | 0 | 2.51±0.22i | 6.52±0.52g | 1.06±0.05g | 5.55±0.27e | 0.14±0.01h | 0.52±0.03g |
| 8 | 30 | 3.12±0.19h | 8.33±0.50f | 1.16±0.07g | 6.19±0.19e | 0.15±0.00h | 0.51±0.02g |
| 8 | 60 | 4.53±0.16f | 8.70±0.48ef | 1.22±0.04g | 5.93±0.20e | 0.13±0.01h | 0.41±0.03h |

**Table 3-1.** Analysis of variance for effects of Selenium (Se) concentration on biochemical attributes of rice seeds.

|  | MDA | | | Starch | | | α-amylase | | | sugar | | |
| --- | --- | --- | --- | --- | --- | --- | --- | --- | --- | --- | --- | --- |
|  | df | F value | P value | df | F value | P value | df | F value | P value | df | F value | P value |
| Se | 2 | 20.442 | 0.0000 | 2 | 16.560 | 0.0000 | 2 | 20.635 | 0.0000 | 2 | 5.512 | 0.0065 |

**Table 3-2.** Effects of Selenium (Se) concentration on biochemical attributes of rice seeds. One unit of the enzyme's activity is the amount of enzyme which released 1 μmol of maltose by 1 mL original enzyme solution in 1 min. Means (n = 5) with different letters differ significantly at the 5% probability level based on Tukey's test.

| Se | MDA | Starch | α-amylase | sugar |
| --- | --- | --- | --- | --- |
|  | (nm∙g-1 seed) | (% DW) | (unitsa) | (% DW) |
| 0 | 11.27±1.44a | 50.24±3.50a | 8.35±0.95c | 7.17±0.36b |
| 30 | 9.90±1.87b | 46.68±4.41b | 9.15±0.96b | 7.34±0.53b |
| 60 | 8.44±0.57c | 43.77±2.51c | 9.98±0.32a | 7.65±0.49a |

**Table 4-1.** Analysis of variance for effects of Selenium (Se) concentration and flooding duration on biochemical attributes of rice seedlings. FD: flooding duration

|  | SOD | | | POD | | | CAT | | | GPx | | | Protein | | | Total chlorophyll | | |
| --- | --- | --- | --- | --- | --- | --- | --- | --- | --- | --- | --- | --- | --- | --- | --- | --- | --- | --- |
|  | df | F value | P value | df | F value | P value | df | F value | P value | df | F value | P value | df | F value | P value | df | F value | P value |
| FD | 3 | 41.400 | 0.0000 | 3 | 205.600 | 0.0000 | 3 | 123.638 | 0.0000 | 3 | 10.756 | 0.0000 | 3 | 264.445 | 0.0000 | 3 | 313.270 | 0.0000 |
| Se | 2 | 400.710 | 0.0000 | 2 | 2458.852 | 0.0000 | 2 | 2686.850 | 0.0000 | 2 | 169.853 | 0.0000 | 2 | 712.536 | 0.0000 | 2 | 238.307 | 0.0000 |
| FD × Se | 6 | 24.517 | 0.0000 | 6 | 26.346 | 0.0000 | 6 | 47.915 | 0.0000 | 6 | 1.805 | 0.1180 | 6 | 4.282 | 0.0016 | 6 | 12.625 | 0.0000 |

**Table 4-2.** Effects of Selenium (Se) concentration and flooding duration on biochemical attributes of rice seedlings. FD: flooding duration. SOD: superoxide dismutase, POD: peroxidase, CAT: catalase, GPx: glutathione peroxidase. Means (n = 5) with different letters differ significantly at the 5% probability level based on Tukey's test.

| FD | Se | SOD | POD | CAT | GPx | Protein | Total chlorophyll |
| --- | --- | --- | --- | --- | --- | --- | --- |
|  |  | (units∙g-1 protein) | (μmol∙min-1∙g-1 protein) | (μmol∙min-1∙g-1 protein) | (μmol∙min-1∙g-1 protein) | (mg∙g-1 FW) | (mg∙g-1 FW) |
| 0 | 0 | 565.38±19.80g | 1.04±0.03j | 0.66±0.01i | 152.18±5.21fg | 0.27±0.01f | 3.32±0.08f |
| 0 | 30 | 663.74±16.60cd | 1.68±0.05g | 1.32±0.03f | 199.03±15.05de | 0.36±0.01c | 4.32±0.07b |
| 0 | 60 | 673.99±55.55cd | 2.25±0.09f | 1.66±0.04d | 289.77±13.82a | 0.44±0.01a | 5.06±0.21a |
| 2 | 0 | 612.50±27.39ef | 1.23±0.05i | 0.73±0.04i | 176.08±7.77ef | 0.24±0.02g | 3.23±0.12f |
| 2 | 30 | 682.47±50.76cd | 2.36±0.08de | 1.48±0.07e | 231.87±10.83bc | 0.35±0.01c | 4.28±0.22b |
| 2 | 60 | 923.54±49.13a | 2.77±0.05b | 2.49±0.07a | 307.70±15.62a | 0.41±0.02b | 5.01±0.41a |
| 4 | 0 | 587.32±27.17fg | 1.42±0.05h | 0.68±0.05i | 122.54±69.06g | 0.20±0.01h | 2.79±0.16g |
| 4 | 30 | 708.49±16.45c | 2.41±0.06d | 1.21±0.05g | 211.88±13.36cd | 0.30±0.01e | 3.73±0.08d |
| 4 | 60 | 933.46±21.75a | 2.90±0.12a | 2.24±0.10b | 280.34±6.19a | 0.36±0.01c | 4.01±0.25c |
| 8 | 0 | 457.42±6.40h | 0.93±0.04k | 0.50±0.03j | 137.15±5.96g | 0.16±0.01i | 2.09±0.10h |
| 8 | 30 | 645.79±18.68de | 2.30±0.09ef | 1.01±0.02h | 197.60±11.11de | 0.21±0.01h | 2.32±0.12h |
| 8 | 60 | 877.01±44.14b | 2.66±0.07c | 1.98±0.13c | 243.53±16.61b | 0.32±0.02d | 2.61±0.19g |

**Table 5-1.** Analysis of variance for effects of Selenium (Se) concentration and flooding duration on rice seedlings Se concentration. FD: flooding duration

|  | Total Se | | | Organic Se | | |
| --- | --- | --- | --- | --- | --- | --- |
|  | df | F value | P value | df | F value | P value |
| FD | 3 | 5.326 | 0.0030 | 3 | 95.491 | 0.0000 |
| Se | 2 | 6982.631 | 0.0000 | 2 | 6908.092 | 0.0000 |
| FD × Se | 6 | 1.622 | 0.1616 | 6 | 32.201 | 0.0000 |

**Table 5-2.** Effects of Selenium (Se) concentration and flooding duration on rice seedlings Se concentration. FD: flooding duration. Means (n = 5) with different letters differ significantly at the 5% probability level based on Tukey's test.

| FD | Se | Total Se | Organic Se |
| --- | --- | --- | --- |
|  |  | mg∙kg^-1^ | mg∙kg^-1^ |
| 0 | 0 | 0.165±0.002d | 0.082±0.002h |
| 0 | 30 | 1.506±0.068c | 0.828±0.024e |
| 0 | 60 | 2.653±0.069a | 1.746±0.062a |
| 2 | 0 | 0.151±0.003d | 0.071±0.003h |
| 2 | 30 | 1.499±0.029c | 0.814±0.061e |
| 2 | 60 | 2.545±0.084b | 1.62±0.044b |
| 4 | 0 | 0.13±0.002d | 0.063±0.004h |
| 4 | 30 | 1.468±0.048c | 0.725±0.029f |
| 4 | 60 | 2.481±0.123b | 1.446±0.039c |
| 8 | 0 | 0.101±0.003d | 0.051±0.003h |
| 8 | 30 | 1.49±0.059c | 0.664±0.031g |
| 8 | 60 | 2.494±0.108b | 1.271±0.070d |

**Table 6-1.** Analysis of variance for effects of Selenium (Se) concentration and flooding duration on total dry weight, yield and Se content in polish rice in 2018-2019. FD: flooding duration

|  | 2018 | | | | | | | | | 2019 | | | | | | | | |
| --- | --- | --- | --- | --- | --- | --- | --- | --- | --- | --- | --- | --- | --- | --- | --- | --- | --- | --- |
|  | Total dry weight | | | Yield | | | Se content | | | Total dry weight | | | Yield | | | Se content | | |
|  | df | F value | P value | df | F value | P value | df | F value | P value | df | F value | P value | df | F value | P value | df | F | p |
| FD | 3 | 29.083 | 0.0000 | 3 | 25.523 | 0.0000 | 3 | 10.073 | 0.0000 | 3 | 24.981 | 0.0000 | 3 | 24.268 | 0.0000 | 3 | 23.480 | 0.0000 |
| Se | 2 | 11.345 | 0.0001 | 2 | 21.269 | 0.0000 | 2 | 3.240 | 0.0479 | 2 | 10.276 | 0.0002 | 2 | 34.530 | 0.0000 | 2 | 11.232 | 0.0001 |
| FD × Se | 6 | 5.355 | 0.0003 | 6 | 5.795 | 0.0001 | 6 | 1.876 | 0.1044 | 6 | 5.905 | 0.0001 | 6 | 7.751 | 0.0000 | 6 | 3.488 | 0.0061 |

| FD | Se | 2018 | | | 2019 | | |
| --- | --- | --- | --- | --- | --- | --- | --- |
|  |  | Total dry weight | Yield | Se content | Total dry weight | Yield | Se content |
|  |  | t∙ha^-1^ | t∙ha^-1^ | mg∙kg^-1^ | t∙ha^-1^ | t∙ha^-1^ | mg∙kg^-1^ |
| 0 | 0 | 10.30±0.55bc | 5.15±0.21efg | 0.101±0.003d | 10.34±0.66cde | 4.93±0.15f | 0.108±0.003g |
| 0 | 3 | 9.85±0.45cd | 5.06±0.12fgh | 0.106±0.004c | 10.44±0.30cd | 5.11±0.07def | 0.112±0.003f |
| 0 | 6 | 10.11±0.40bc | 5.30±0.19cde | 0.108±0.003abc | 10.35±0.14cde | 5.51±0.21bc | 0.113±0.002ef |
| 2 | 0 | 10.28±0.51bc | 5.13±0.11efg | 0.109±0.004abc | 10.25±0.62de | 5.05±0.14ef | 0.114±0.003def |
| 2 | 3 | 10.66±0.26b | 5.37±0.15bcd | 0.110±0.002abc | 11.02±0.48bc | 5.61±0.12ab | 0.123±0.002a |
| 2 | 6 | 12.01±0.90a | 5.50±0.08bc | 0.111±0.004ab | 11.48±0.88b | 5.67±0.16ab | 0.118±0.002bc |
| 4 | 0 | 10.33±0.68bc | 5.16±0.22efg | 0.107±0.002bc | 10.81±0.26bcd | 5.22±0.18de | 0.117±0.002bcd |
| 4 | 3 | 11.35±0.42a | 5.56±0.15b | 0.106±0.003c | 11.40±0.22b | 5.80±0.24a | 0.121±0.004ab |
| 4 | 6 | 11.67±0.52a | 5.77±0.15a | 0.107±0.004abc | 12.35±0.55a | 5.82±0.14a | 0.116±0.002cde |
| 8 | 0 | 9.38±0.17d | 4.88±0.16h | 0.112±0.002a | 9.70±0.57ef | 5.13±0.13def | 0.119±0.002bc |
| 8 | 3 | 9.92±0.37bcd | 5.25±0.05def | 0.108±0.005abc | 10.79±0.63bcd | 5.33±0.21cd | 0.119±0.003bc |
| 8 | 6 | 9.59±0.53cd | 4.96±0.15gh | 0.111±0.003ab | 9.49±0.33f | 4.98±0.26ef | 0.116±0.003cde |

**Table 6-2.** Effects of Selenium (Se) concentration and flooding duration on total dry weight, yield and Se content in polish rice in 2018-2019. FD: flooding duration. Means (n = 5) with different letters differ significantly at the 5% probability level based on Tukey's test.
